# CF750-A33scFv-Fc-Based Optical Imaging of Subcutaneous and Orthotopic Xenografts of GPA33-Positive Colorectal Cancer in Mice

**DOI:** 10.1155/2015/505183

**Published:** 2015-05-21

**Authors:** Danfeng Wei, Qing Fan, Huawei Cai, Hao Yang, Lin Wan, Lin Li, Xiaofeng Lu

**Affiliations:** ^1^Key Laboratory of Transplant Engineering and Immunology, Regenerative Medical Center, West China Hospital, Sichuan University, Chengdu 610041, China; ^2^Department of Nuclear Medicine, West China Hospital, Sichuan University, Chengdu 610041, China

## Abstract

Antibody-based imaging agents are attractive as adjuvant diagnostic tools for solid tumors. GPA33 is highly expressed in most human colorectal cancers and has been verified as a diagnostic and therapeutic target. Here, we built an A33scFv-Fc antibody against GPA33 by fusing A33scFv to the Fc fragment of human IgG1 antibodies. The A33scFv-Fc specifically binds GPA33-positive colorectal cancer cells and tumor tissues. After the intravenous injection of mice bearing subcutaneous GPA33-positive LS174T tumor grafts with near-infrared fluorescence probe CF750-labeled A33scFv-Fc (CF750-A33scFv-Fc), high contrast images of the tumor grafts could be kinetically documented within 24 h using an optical imaging system. However, GPA33-negative SMMC7721 tumor grafts could not be visualized by injecting the same amount of CF750-A33scFv-Fc. Moreover, in subcutaneous LS174T tumor-bearing mice, tissue scanning revealed that the CF750-A33scFv-Fc accumulated in the tumor grafts, other than the kidney and liver. In mice with orthotopic tumor transplantations, excrescent LS174T tumor tissues in the colon were successfully removed under guidance by CF750-A33scFv-Fc-based optical imaging. These results indicate that CF750-A33scFv-Fc can target GPA33, suggesting the potential of CF750-A33scFv-Fc as an imaging agent for the diagnosis of colorectal cancer.

## 1. Introduction 

Colorectal cancer is one of the most common malignancies in the world, as the third most common cancer in men and the second in women [1]. Although colorectal cancer incidence rates are stabilizing or even declining in historically high-risk areas (United States, New Zealand, and Canada), they are rapidly increasing in several historically low-risk countries (China, Japan, Korea, and Eastern European countries) [2, 3]. Colorectal cancer mainly results from a series of genetic changes leading to the progressive and irreversible loss of the normal control of cell growth and differentiation [4]. In addition, several environmental factors mostly related to diet and lifestyle have been identified and seem to play a certain role in the development of colorectal cancer [5].

The development of colorectal cancer has been revealed as an ordered process spanning three main phases: initiation, promotion, and progression [6]. Clinical data from colorectal cancer in high-resource countries have demonstrated that the mortality of colorectal cancer can be reduced by early treatment [7–9]. Consequently, early diagnosis plays important role in reducing the burden of colorectal cancer. However, the current diagnosis of colorectal cancer is limited by fecal occult blood tests (FOBTs), flexible sigmoidoscopy, and colonoscopy [8, 10]. As a result, it is urgent to develop novel tools for the early diagnosis of colorectal cancer.

Noninvasive molecular imaging has become popular in recent years for the diagnosis of solid tumors [11, 12]. Antibodies against tumor cell-specific surface markers are ideal for tumor imaging because of their high specificity and affinity for antigens [13]. GPA33, a 43 kDa membrane glycoprotein, is highly expressed in over 95% of human colorectal cancers [14]. In addition, no circulating GPA33 antigen has been detected [15]. These results suggest that GPA33 might be a candidate marker for the diagnosis and therapy of colorectal cancer. Consequently, many murine antibodies and their humanized antibodies against GPA33 have been developed in the past decades. Of these antibodies, A33 (a murine monoclonal antibody against GPA33) [16] and huA33 (the humanized A33) [17] have been widely used as imaging tools for mice with human colon cancer xenografts and for colon cancer patients [16, 18, 19]. However, these antibodies are limited by their low affinity for GPA33 [20].

It was well known that the affinity of rabbit antibodies is higher than that of murine antibodies. Consequently, a rabbit antibody against GPA33 has been developed and humanized [20]. As expected, the affinity of this humanized rabbit antibody against GPA33 (hurA33) was much higher than that of the murine antibody A33. A HurA33-derived single chain fragment of variable antibody (A33scFv) was recently developed for use in drug delivery [21, 22]. Considering the slow tumor targeting of large hurA33 and the low affinity of small A33scFv, it is better to develop divalent diabodies, minibodies, or scFv-Fc antibodies against GPA33 as imaging tools. In addition, taking into account the risk of radiation injury by radioactive antibodies, it is urgent to develop nonradioactive probes for antibody labeling. In fact, optical tumor imaging with near-infrared (NIR) fluorescence probe-labeled antibodies has become more and more popular in recent years [23, 24].

In this paper, we first produced a divalent antibody, A33scFv-Fc, against GPA33 by fusing the humanized A33scFv to the Fc fragment of hIgG1 antibodies. Subsequently, we determined the immunoreactivity of CF750-, ^131^I-, and FITC-labeled A33scFv-Fc. Moreover, we evaluated CF570-A33scFv-Fc by optically imaging mice with subcutaneous xenografts of human colon cancer. We also performed orthotopic tumor tissue dissections under the guidance of optical imaging with CF750-A33scFv-Fc. Finally, we analyzed the biodistribution of ^131^I-labeled A33scFv-Fc in a subcutaneous xenograft mouse model.

## 2. Materials and Methods

### 2.1. Construction of a pPIC9K-A33scFv-Fc Expression Plasmid

The genes encoding the variable heavy (VH) and light (VL) chains of intact antibody against A33 antigen were designed based on their amino acid sequences [20]. The single chain fragment of variable antibody against A33 antigen (A33scFv) was constructed in the format VH-(Gly4Ser)3-VL, with an additional 6His tag at the N-terminus.* Eco*RI and* Avr*II restriction sites were added at the 5′ and 3′ ends of the gene encoding A33scFv, which was synthesized by Genscript Corporation (Nanjing, China). To construct the expression plasmid for A33scFv-Fc, an* Eco*RI/*Avr*II double-digested A33scFv gene was ligated to a pPIC9K-hIgG1Fc plasmid containing a gene encoding an hIgG1 Fc fragment [25]. The sequence-verified plasmid was designated as pPIC9K-A33scFv-Fc.

### 2.2. Expression and Purification of the A33scFv-Fc

To express the A33scFv-Fc, we linearized the plasmid of pPIC9K-A33scFv-Fc using* Sal*I restriction enzymes, transforming them into* Pichia pastoris *GS115 competent cells by electroporation (1.5 kV, 200 *Ω*, and 25 *μ*F) according to the instructions for the* P. pastoris* expression kit (Invitrogen, CA, USA). His^+^ transformants were selected on histidine-deficient minimal dextrose (MD) plates. Subsequently, positive colonies were screened through PCR using the *α*-factor primer (5′-TACTATTGCCAGCATTGCTGC-3′) and the 3′ AOX1 primer (5′-GCAAATGGCATTCTGACATCC-3′). Moreover, the A33scFv-Fc-producing colonies were identified by western blot with an anti-His tag antibody. The expression and purification of the A33scFv-Fc were performed similarly, according to our previous work [26]. Briefly, cells derived from a single colony were inoculated into 25 mL buffered glycerol-complex medium (BMGY) and incubated at 28°C with shaking (260 rpm) overnight. Then, the culture was transferred into 1 L fresh BMGY, and all of the cells were collected by centrifugation at room temperature (3,500 g for 5 min) until the *A*_600 nm_ of the culture reached approximately 6, at which point it was resuspended in 100 mL of buffered methanol-complex medium (BMMY). Methanol (3%) was added to the media daily to induce the expression of the A33scFv-Fc antibody. The culture supernatant was collected by centrifugation at 4°C (15,000 g for 15 min) after an induction period, and the supernatant was dialyzed against binding buffer (50 mM Tris-HCl, 0.5 M NaCl, and 10 mM imidazole, pH 8.0) at 4°C overnight. To improve the yield ofantibody, the expression conditions, including the initial pH values of the medium and the induction time, were optimized. The A33scFv-Fc was purified using Ni-NTA superflow (Qiagen, CA, USA). Subsequently, the purified antibody was dialyzed against phosphate-buffered saline (PBS, 8 g L^−1^ NaCl, 0.2 gL^−1^ KCl, 3.49 gL^−1^ Na_2_HPO_4_·12H_2_O, and 0.2 gL^−1^ KH_2_PO_4_) at 4°C overnight. The protein concentration was measured using the Bradford method.

### 2.3. SDS-PAGE and Western Blot

Sodium dodecyl sulfate polyacrylamide gel electrophoresis (SDS-PAGE) and western blots were performed according to the descriptions in our previous work [27], with some modifications. Briefly, the proteins were separated on 10% gels and visualized by Coomassie Brilliant Blue. For western blots, the separated proteins were transferred onto polyvinylidene difluoride (PVDF) membranes (Bio-Rad, CA, USA) and incubated with a horseradish peroxidase- (HRP-) labeled antibody against the 6-His tag (Qiagen, CA, USA). Finally, the target proteins were visualized using a chemiluminescent substrate for HRP detection (Thermo, IL, USA).

### 2.4. Labeling of A33scFv-Fc

#### 2.4.1. Labeling of A33scFv-Fc with FITC

The labeling of the antibody with fluorescein isothiocyanate (FITC) was performed according to the EZ-Label FITC protein labeling kit (Pierce, CA, USA). Briefly, a 24-fold molar excess of FITC was added to the antibody (1 mg/mL, pH 8.5). After incubation at room temperature for 1 h in the darkness, the mixture was dialyzed against PBS with several buffer changes to remove the unconjugated FITC.

#### 2.4.2. Labeling of A33scFv-Fc with CF750

The labeling of the antibody with CF750 was performed according to our previous work [26]. Briefly, CF750 dye dissolved in dimethyl sulfoxide (DMSO) was added to the antibody (1 mg/mL, pH 8.3) at a 12 : 1 molar ratio of dye to antibody. After incubation at room temperature for 1 h in darkness, the mixture was dialyzed against PBS with several buffer changes to remove the excessive CF750 dye. The degree of labeling (DOL) was calculated according to the following formula: DOL = (*A*_755 nm_  ×  molecular weight of antibody × dilution factor)/(*ε*  × concentration of antibody). The molar extinction coefficient (*ε*) of CF750 is 250,000.

#### 2.4.3. Labeling of A33scFv-Fc with Radionuclide ^131^I

The labeling of the antibody with radionuclide ^131^I was described in our previous work [26]. Briefly, the mixture containing 50 *μ*g antibody (1 mg/mL), 7.4 MBq of ^131^I-labeled sodium iodide (specific radioactivity ≥ 2.8 MBq/*μ*L), and 7.5 *μ*g of N-bromosuccinimide (1 mg/mL) was incubated at room temperature with shaking for 5 min. A PB-10 desalting column was used to remove the free sodium iodide, and thin layer chromatography (TLC) was used to determine the degree of labeling (DOL).

### 2.5. Cell Culture

The GPA33 antigen-positive LS174T, COLO205 cells and GPA33 antigen-negative SMMC7721, AGS cells were all purchased from the American Type Culture Collection (ATCC, VA, USA). Cells were cultured in medium (Dulbecco's Modified Eagle Medium for LS174T, RPMI-1640 for COLO205 and SMMC7721, and F-12K for AGS) containing 10% fetal bovine serum (FBS) at 37°C in a humidified atmosphere containing 5% CO_2_.

### 2.6. Immunoreactivity Assays

#### 2.6.1. Immunoreactivity Assay for FITC-A33scFv-Fc

To evaluate the immunoreactivity of FITC-labeled A33scFv-Fc, approximately 3 × 10^5^ cells were incubated with 20 nM FITC-labeled antibody in 100 *μ*L PBS containing 0.5% calf serum at 37°C for 1 h in darkness, followed by analysis using a flow cytometer (Cytomics FC 500, Beckman Coulter, CA, USA). A FITC-labeled isotype antibody was utilized as a control in these assays.

#### 2.6.2. Immunoreactivity Assay for CF750-A33scFv-Fc

The immunoreactivity of CF750-A33scFv-Fc was analyzed by dose-dependent cell binding assays. Briefly, approximately 3 × 10^5^ LS174T cells were incubated with CF750-A33scFv-Fc antibody at different concentrations (1, 2, 4, 10, and 20 nM) in 100 *μ*L PBS containing 0.5% calf serum at 37°C for 1 h in darkness. After two washes with PBS containing 0.5% calf serum, the cells were transferred onto a 96-well plate and scanned by the IVIS optical imaging system (Caliper Life Sciences, CA, USA). The fluorescence signal intensities of cells were analyzed.

#### 2.6.3. Immunoreactivity Assay for ^131^I-A33scFv-Fc

Dose-dependent cell binding assays were used to evaluate the immunoreactivity of the ^131^I-labeled antibody. Briefly, approximately 6 × 10^5^ LS174T cells were incubated with the ^131^I-A33scFv-Fc antibody at different concentrations (1, 2.5, 5, 10, 20, 30, and 60 nM) in 200 *μ*L PBS containing 0.5% calf serum at 37°C for 1 h. After incubation, the cells were washed with PBS containing 0.5% calf serum twice, and the total radioactivity of the bound antibody was counted using a FJ-2008PS Gamma counter (Xi'an Nuclear Instrument Factory, Shanxi, China). The free ^131^I+A33-scFv-Fc was used as a control.

### 2.7. Immunofluorescence Histochemistry

The tumor xenografts and liver tissues obtained from mice were immediately cut into frozen sections and stained with the FITC-labeled antibody in the dark at 4°C overnight. The cell nuclei were visualized using DAPI before being observed under fluorescence microscopy. A FITC-labeled isotype antibody was utilized as a negative control.

### 2.8. Optical Imaging of Mice Bearing Subcutaneous Tumor Xenografts

All of the protocols used in this experiment were approved by the University Animal Care and Use Committee. LS174T, COLO205, or SMMC7721 (1 × 10^6^ cells/mouse) were subcutaneously implanted at the right flank of female BALB/C nu/nu mice (4~6 weeks). Tumor growth was monitored every day, and tumor volumes were calculated according to the following formula: width^2^  × length × 0.5. To monitor the tumor uptake of antibody, the mice (*n* = 3) bearing subcutaneous tumor xenografts (100~200 mm^3^) were intravenously injected with CF750-A33scFv-Fc (100 *μ*g in 100 *μ*L) and were followed by kinetic scans using the IVIS optical imaging system at different time points after injection. To compare the uptake of CF750-A33scFv-Fc by the tumor and the other organs/tissues, the injected mice (*n* = 3) bearing LS174T xenografts were sacrificed, and the tumor, heart, liver, spleen, lung, kidney, stomach, colon, pancreas, small intestine, brain, and muscle were scanned at different time points after injection. The fluorescence signal intensities in the tumor and the other organs/tissues were analyzed. In the control group, the mice were injected with the same amount of unconjugated CF750 dye.

### 2.9. Optical Imaging of Mice with Orthotopic Tumor Transplantations

LS174T cells (1 × 10^6^ cells/mouse) were subcutaneously injected into female BALB/C nu/nu mice (4~6 weeks). When the tumor volume reached approximately 200~300 mm^3^, the tumor grafts were dissected and immediately cut into small granules (~l mm^3^), followed by the transplantation of these tissues into the colons of other nude mice. Once the tumor grafts were palpable, the mice (*n* = 3) were injected with CF750-A33scFv-Fc (100 *μ*g in 100 *μ*L) followed by imaging using the IVIS optical imaging system at 5 h after injection. Subsequently, the mice were sacrificed, and laparotomy was performed. The orthotopic tumor tissues were identified and dissected under guidance by CF750-A33scFv-Fc antibody-based optical imaging.

### 2.10. Biodistribution of ^131^I-A33scFv-Fc

To evaluate the biodistribution of ^131^I-A33scFv-Fc, mice (*n* = 3) bearing subcutaneous LS174T tumor xenografts were intravenously injected with ^131^I-A33scFv-Fc (185 kBq/g body weight) and sacrificed at different time points. The tumor grafts and normal organs/tissues were taken out, weighed, and counted for radioactivity using the FJ-2008PS Gamma counter. The results are expressed as the percent injected dose per gram of tissue (%ID/g).

## 3. Results

### 3.1. Production of A33scFv-Fc

**Figure 1:**
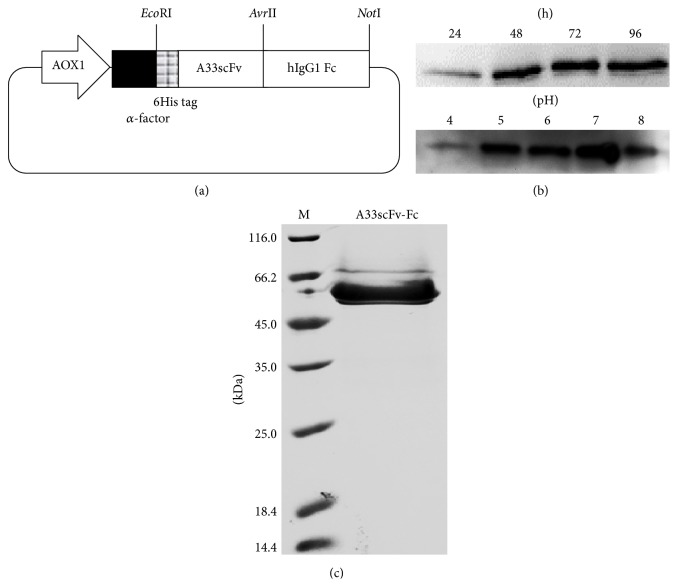
Expression and purification of A33scFv-Fc. (a) Schematic diagram of the pPIC9K-A33scFv-Fc expression vector. (b) Effects of the initial pH (4~8) of the inductive medium and inductive time (24~96 h) on the production of A33scFv-Fc. (c) SDS-PAGE analysis of purified A33scFv-Fc (6 *μ*g). M: protein markers.

To construct the pPIC9K-A33scFv-Fc expression plasmid, an* Eco*RI/*Avr*II double digested A33scFv gene was ligated to the pPIC9K-hIgG1Fc vector. The schematic diagram of the pPIC9K-A33scFv-Fc expression plasmid is shown in Figure 1(a). To produce the A33scFv-Fc antibody, the pPIC9K-A33scFv-Fc plasmid was transformed into* P. pastoris* GS115 competent cells, followed by their identification with PCR and western blot (data not shown). To improve the production of A33scFv-Fc, the initial pH of inductive media and inductive time were optimized. As shown in Figure 1(b), A33scFv-Fc was expressed in media with an initial pH of 4.0~8.0 and a peak at pH 7.0. Moreover, time-dependent analysis demonstrated that A33scFv-Fc accumulated in the medium throughout the induction time of 24~96 h. Finally, the expression of A33scFv-Fc was induced for 96 h by daily additions of 3% methanol to a medium with an initial pH of 7.0.  Figure 1(c) showed that A33scFv-Fc was recovered with approximately 90% purity from the supernatant using Ni-NTA affinity chromatography.

### 3.2. Immunoreactivity of A33scFv-Fc

**Figure 2:**
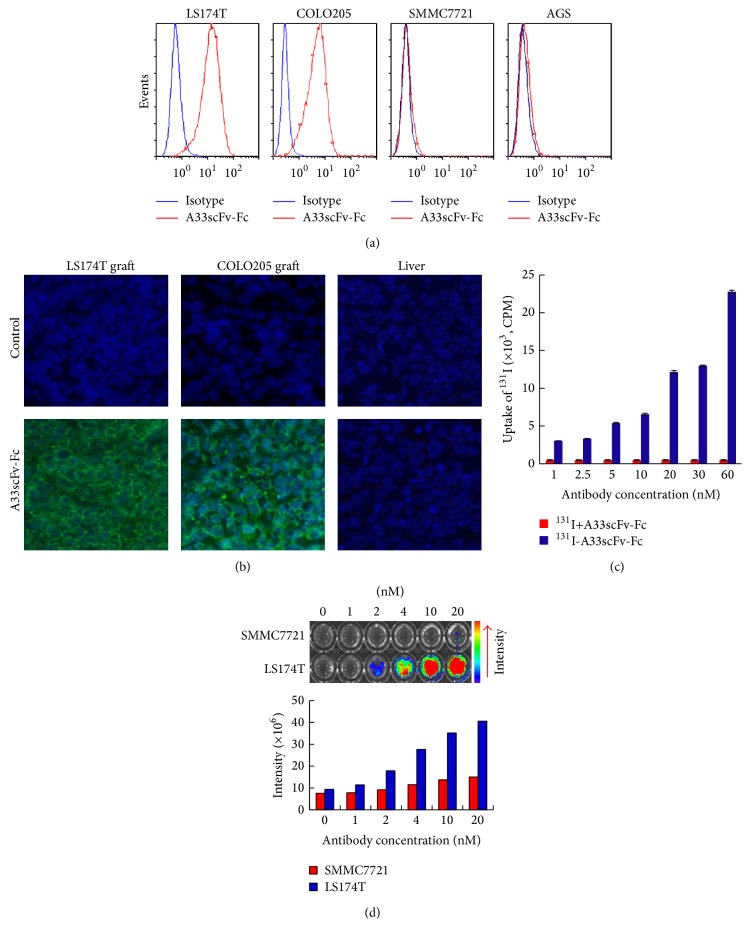
Immunoreactivity and specificity of A33scFv-Fc. (a) Cell binding assays of FITC-labeled A33scFv-Fc. GPA33-positive (LS174T, COLO205) and GPA33-negative (SMMC7721, AGS) cells were incubated with FITC-A33scFv-Fc, followed by flow cytometric analysis. (b) Immunofluorescence chemical assay for the tissue binding of FITC-A33scFv-Fc. LS174T, COLO205 tumor tissues, and liver tissues were stained with FITC-A33scFv-Fc and observed under a fluorescence microscope. The cell nuclei were stained with DAPI, and an isotype antibody was used as a negative control. (c) The cell binding of ^131^I-labeled A33scFv-Fc. LS174T cells were incubated with ^131^I-A33scFv-Fc at different concentrations (1~60 nM), followed by counting the radioactivity of cells using a gamma counter. The mixture containing the same amount of unconjugated ^131^I and A33scFv-Fc was used as a control. (d) Cellular binding of CF750-labeled A33scFv-Fc. SMMC-7721 and COLO205 cells were incubated with the CF750-A33scFv-Fc at the indicated molar concentrations (0~20 nM) and followed by scanning with the IVIS optical imaging system. The fluorescence intensities of the cells were calculated and compared.

The immunoreactivity of the FITC-, CF750-, and ^131^I-labelled A33scFv-Fc was examined using cell binding and immunofluorescence histochemistry. As shown in Figure 2(a), flow cytometry analysis demonstrated that the binding rates of FITC-A33scFv-Fc to GPA33-positive LS174T and COLO205 were 94% and 93.8%, respectively. In contrast, the binding rates of FITC-A33scFv-Fc to GPA33-negative SMMC7721 and AGS cells were as low as 1.0% and 1.7%. A33scFv-Fc has been indicated to be able to specifically bind GPA33-positive cells. Immunofluorescence histochemistry further verified the specificity of A33scFv-Fc for the GPA33 antigen. As shown in Figure 2(b), FITC-A33scFv-Fc bound GPA33-positive LS174T and COLO205 tumor tissues but not GPA33-negative liver tissue.

The immunoreactivity of ^131^I-labeled A33scFv-Fc was also determined by cell binding assays. LS174T cells were incubated with either ^131^I-A33scFv-Fc or free ^131^I+A33-scFv-Fc, followed by counting the cell-bound radioactive signal. As shown in Figure 2(c), after incubation with ^131^I-A33scFv-Fc, the radioactive signals of the cells increased along with the dose of the antibody. In contrast, dose-dependent binding was not observed in cells incubated with free ^131^I+A33-scFv-Fc. These results demonstrate that ^131^I-labeled A33scFv-Fc is active in cell binding.

To examine the immunoreactivity of CF750-labeled antibody, LS174T cells and SMMC7721 cells were incubated with antibodies followed by being transferred into a 96-well plate and scanned using an IVIS optical imaging system.  Figure 2(d) showed that the fluorescence intensity of the LS174T cells increased alongside the antibody. On the other hand, only low level of fluorescence intensity was detected in SMMC7721 cells incubated with the same amount of CF750-A33scFv-Fc. These data indicate that CF750-labeled A33scFv-Fc is able to specifically bind GPA33-positive LS174T cells.

### 3.3. Subcutaneous Tumor Targeting of CF750-A33scFv-Fc

**Figure 3:**
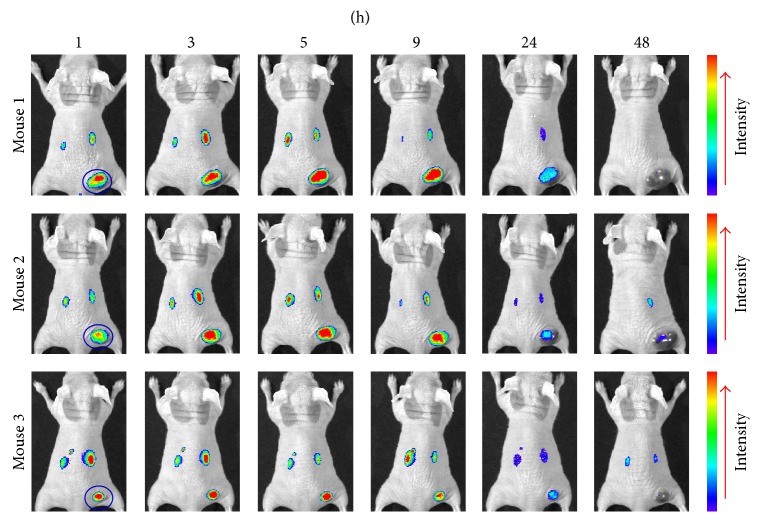
CF750-A33scFv-Fc-mediated optical imaging of mice bearing subcutaneous LS174T xenografts. Mice (*n* = 3) were intravenously injected with CF750-A33scFv-Fc (100 *μ*g) and scanned at 1, 3, 5, 9, 24, and 48 h after injection. The LS174T tumor xenografts are indicated by blue circle.

**Figure 4:**
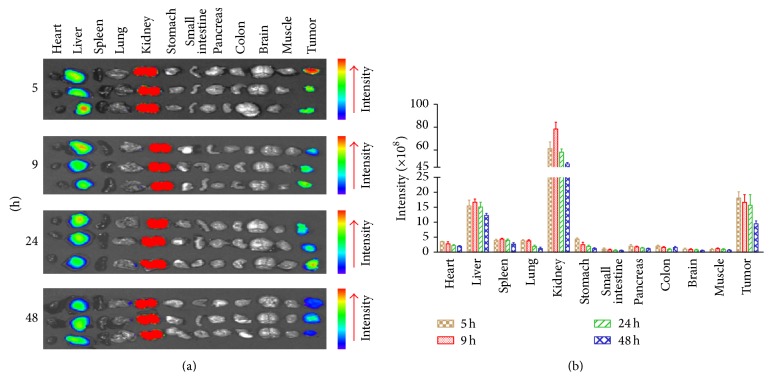
Tissue distribution of CF750-A33scFv-Fc in mice bearing subcutaneous LS174T xenografts. (a) Optical imaging of the tumor grafts and organ tissues at different time points. Mice (*n* = 3) were intravenously injected with CF750-A33scFv-Fc (100 *μ*g) and sacrificed at 5, 9, 24, and 48 h after injection. Tumor grafts and other normal organs/tissues were taken out and scanned using the optical imaging system. (b) The fluorescence intensities of CF750-A33scFv-Fc in the tumor grafts and the organs/tissues at the indicated time points.

To monitor the tumor uptake of the antibody, mice bearing subcutaneous LS174T xenografts were intravenously injected with CF750-A33scFv-Fc followed by kinetic scanning using an optical imaging system at 1, 3, 5, 9, 24, and 48 h after injection. As shown in Figure 3, LS174T tumor grafts were visible at 1 h after injection, indicating the rapid tumor uptake of A33scFv-Fc. Moreover, A33scFv-Fc accumulated in the tumor grafts with time, peaking at 5 h. High-contrast images of the subcutaneous LS174T tumor grafts were obtained at 3~9 h after injection. Although the tumor uptake of A33scFv-Fc was reduced by 24 h, it was still detectable at 48 h after injection. To further compare the antibody uptake, mice injected with CF750-A33scFv-Fc were sacrificed, and the tumor grafts and other organs/tissues were scanned at 5, 9, 24, and 48 h after injection. As shown in Figures 4(a) and 4(b), fluorescence signals were mainly detected in the kidney, liver, and tumor grafts at all time points. The uptake rates of A33scFv-Fc at each time point were as follows (from high to low): kidney > liver > tumor > spleen > lung > heart > stomach > colon > pancreas > small intestine > brain > muscle. The uptake ratios at 5, 9, 24, and 48 h were as follows: tumor-to-muscle: 19.8 ± 5.3, 15.5 ± 2.5, 17.4 ± 5.6, and 17.0 ± 5.1; tumor-to-colon: 9.1 ± 1.9, 11.0 ± 0.2, 13.4 ± 1.5, and 7.8 ± 3.5; and tumor-to-small intestine: 19.1 ± 7.3, 21.8 ± 4.8, 24.6 ± 5.5, and 20.8 ± 2.1. These results demonstrated the high persistence of CF750-A33scFv-Fc in GPA33-positive LS174T tumor xenografts.

**Figure 5:**
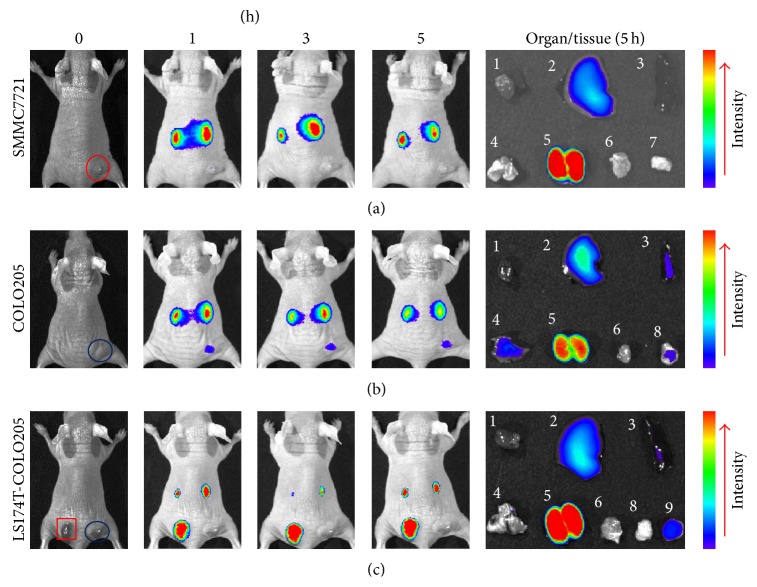
Optical imaging of mice bearing single SMMC7721 (a), COLO205 (b), or dual LS174T and COLO205 (c) xenografts. Mice (*n* = 3) bearing tumor grafts were intravenously injected with CF750-A33scFv-Fc (100 *μ*g) and scanned at 0, 1, 3, and 5 h after injection. SMMC7721, COLO205, and LS174T xenografts are indicated with red circle, blue circle, and red square, respectively. After the last scans (5 h), the mice were sacrificed, and the organs/tissues were removed and scanned. 1: heart, 2: liver, 3: spleen, 4: lung, 5: kidney, 6: muscle, 7: SMMC7721 tumor graft, 8: COLO205 tumor graft, and 9: LS174T tumor graft.

However, injecting the same amount of CF750-A33scFv-Fc did not allow the visualization of the subcutaneous GPA33-negative SMMC 7721 tumor grafts (Figure 5(a)), indicating the high specificity of A33scFv-Fc. Surprisingly, we failed to obtain high-contrast images of mice with GPA33-positive COLO205 tumor grafts after they had been injected with the same amount of CF750-A33scFv-Fc (Figure 5(b)). To further compare the antibody uptakes by the COLO205 and LS174T xenografts, we inoculated COLO205 and LS174T cells in the same mice. As shown in Figure 5(c), after the injection of CF750-A33scFv-Fc into mice with dual tumor grafts, the fluorescence signal in the COLO205 xenograft was significantly lower than that in the LS174T xenograft. Because both COLO205 and LS174T cells were GPA33-positive, these results suggest that CF750-A33scFv-Fc might not be effectively delivered into COLO205 tumor grafts.

### 3.4. Orthotopic Tumor Targeting of CF750-A33scFv-Fc

**Figure 6:**
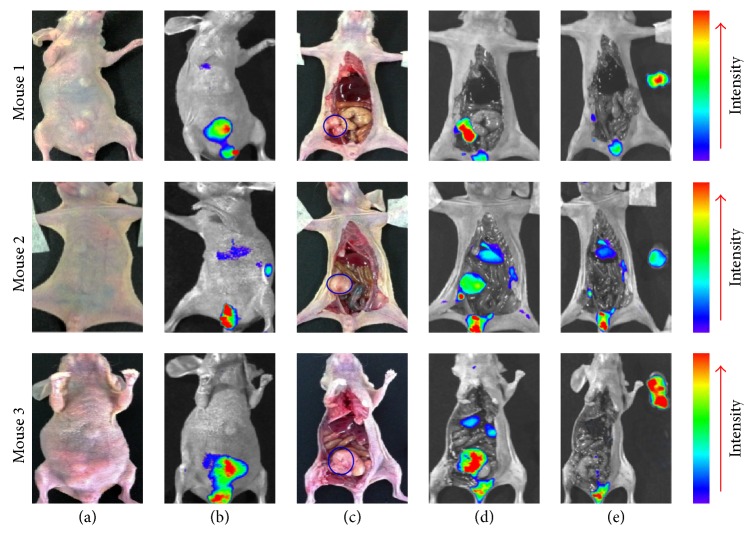
CF750-A33scFv-Fc-based optical imaging-directed orthotopic tumor tissue dissection. LS174T tumor tissues derived from xenografts were inoculated into colons of mice. Once the tumor tissues were palpable, the mice (*n* = 3) were injected with CF750-A33scFv-Fc (100 *μ*g) followed by scanning and tumor tissue dissection at 5 h after injection. Before laparotomy, the mice were first imaged using an optical imaging system with white light (a) and NIR filter settings (b). Next, laparotomy was performed, and the excrescent tumor tissues in the colon were identified by an optical imaging system (c and d). Finally, the orthotopic tumor tissues were removed under the guide of optical imaging (e). LS174T xenografts are indicated with blue circle.

To evaluate the targeting of CF750-A33scFv-Fc in an orthotopic LS174T tumor transplantation mice model, the injected mice were first imaged using the optical imaging system from the abdomen without laparotomy. As shown in Figures 6(a) and 6(b), the bladders of three mice were visible under the optical imaging system at 5 h after injection, but tumor grafts were palpable in only two mice. Once the laparotomy was performed, excrescent tumor tissues in the colon were distinguished from normal tissues in three mice using CF750-A33scFv-Fc (Figures 6(c) and 6(d)). Moreover, tumor tissues were accurately dissected under the guidance of an IVIS optical imaging system (Figure 6(e)). These results demonstrate that the CF750-A33scFv-Fc can target orthotopic LS174T tumor grafts.

### 3.5. Biodistribution Analysis of ^131^I-A33scFv-Fc

**Figure 7:**
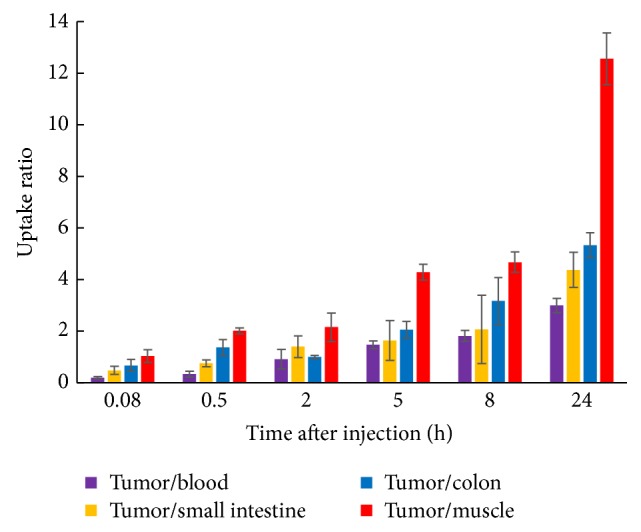
The ^131^I-A33scFv-Fc uptake ratio of tumor to normal tissues at different time points. Mice (*n* = 3) bearing subcutaneous LS174T tumor xenografts were intravenously injected with ^131^I-A33scFv-Fc (185 kBq/g body weight) and then sacrificed at 0.08, 0.5, 2, 5, 8, and 24 h after injection, respectively. The radioactivity of the organ/tissue was counted, and the ratios of tumor-to-blood, tumor-to-small intestine, tumor-to-colon, and tumor-to-muscle were calculated.

In a biodistribution assay, mice bearing subcutaneous LS174T tumor xenografts (300~400 mm^3^) were intravenously injected with ^131^I-A33scFv-Fc. Subsequently, the mice were sacrificed at 0.08, 0.5, 2, 5, 8, and 24 h after injection, followed by counting the radioactivity of tumor and organs/tissues. As shown in Table 1, the radioactivity of ^131^I-A33scFv-Fc in the tumor at 5 min was 4.12 ± 0.76%, indicating a rapid tumor uptake that increased with time and peaked at 5 h after injection, with an activity of 5.45 ± 1.25%. Thereafter, the tumor's radioactivity began to decrease but persisted at a high level at 24 h after injection. The radioactivity in the tumor was 2.03 ± 0.51% at 24 h after injection, compared to 0.68 ± 0.19%, 0.16 ± 0.05%, 0.47 ± 0.16%, and 0.38 ± 0.11% in the blood, muscle, small intestine, and colon, respectively. As shown in Figure 7, the ratios of tumor-to-muscle were 4.3 ± 0.3, 4.7 ± 0.4, and 12.6 ± 1 at 5, 8, and 24 h after injection, respectively. The ratio of tumor-to-blood increased from 1.5 ± 0.1 at 5 h to 3.0 ± 0.3 at 24 h. In addition, the ratios of tumor-to-small intestine and tumor-to-colon were as high as 4.4 ± 0.7 and 5.3 ± 0.5, respectively, at 24 h after injection. These results demonstrate that the ^131^I-A33scFv-Fc was able to specifically localize at the LS174T tumor.

## 4. Discussion

In this study, divalent A33scFv-Fc against GPA33 was prepared by fusing A33scFv to the Fc fragment of an hIgG1 antibody.* In vitro* cell binding assays demonstrated that the A33scFv-Fc produced by* P. pastoris* can bind GPA33-positive but not GPA-negative tumor cells. In mice bearing subcutaneous xenografts, CF750-labeled A33scFv-Fc was accumulated rapidly in GPA33-positive LS174T xenografts but not in GPA33-negative SMMC7721 xenografts. Moreover, orthotopic LS174T tumor tissues were dissected successfully under the guidance of an optical imaging system using CF750-labeled A33scFv-Fc as an indicator. Biodistribution analysis revealed a rapid tumor uptake and a high level persistence of A33scFv-Fc in the xenografts. These results suggest that A33scFv-Fc could be developed as a useful imaging agent for colorectal cancer.

Due to their high specificity and affinity for antigens, antibody-based imaging agents are ideal for tumor diagnostics. Taking into account the slow tumor targeting and serum persistence of large intact antibodies, approaches for building small antibodies, including scFv-Fc, minibody, diabody, and scFv, have been constructed in the past decades [28, 29]. Building scFv was the first step toward engineering a small antibody. In fact, we began by preparing A33scFv and evaluating its potential as an imaging agent for GPA33-positive colorectal cancer. As shown in supplementary Figure 1(a) (in Supplementary Material available online at http://dx.doi.org/10.1155/2015/505183), A33scFv can also bind GAP33-positive LS174T cells. However, the affinity of A33scFv-Fc for LS174T cells was much higher than that of A33scFv. For example, the mean fluorescence intensities (MFI) of cells incubated with 10 and 20 nM FITC-A33scFv-Fc were 4.5- and 4.2-fold of that of A33scFv, respectively (supplementary Figure 1(b)). Moreover, the injection of CF750-A33scFv-Fc produced high-contrast images of mice bearing LS174T xenografts (Figure 3), but the injection of the same amount of CF750-A33scFv gave images with low contrast (supplementary Figure 2(a)). Further tissue scanning demonstrated that the tumor uptake of CF750-A33scFv-Fc (Figure 4(a)) was obviously higher than that of CF750-A33scFv (supplementary Figure 2(b)). The fluorescence intensity of CF750-A33scFv-Fc in tumor grafts at 9 h (Figure 4(b)) was approximately 3~5 times higher than that of CF750-A33scFv at 7 h (supplementary Figure 2(c)). In addition, the CF750-A33scFv-Fc uptake ratios of tumor-to-muscle, tumor-to-colon, and tumor-to-small intestine were 15.5 ± 2.5, 11.0 ± 0.2, and 21.8 ± 4.8, respectively, compared to 7.5 ± 1.9, 3.2 ± 1.5, and 5.4 ± 2.1 among CF750-A33scFv. These results suggest that divalent CF750-A33scFv-Fc is more properly used than monovalent CF750-A33scFv as an imaging agent for colorectal cancer. Nevertheless, because an Fc fragment might introduce such side effects as antibody-dependent cell-mediated cytotoxicity (ADCC) and complement-dependent cytotoxicity (CDC) [30], it might be better to produce a GPA33-recognizing diabody or minibody without an Fc fragment as an imaging agent in the future.

It is known that NIR fluorescence probes have low autofluorescence and low absorbance in normal tissue within the NIR region (700~900 nm), which can potentially increase the sensitivity and specificity of a cancer diagnosis [31]. Consequently, NIR fluorescence probe-labeled antibodies without radiation injury have become attractive as novel tools for tumor imaging. Usually, mice bearing subcutaneous tumor xenografts are used for the preclinical biological evaluation of tumor imaging agents. In our experiment, we first evaluated CF750-A33scFv-Fc in mice bearing subcutaneous LS174T tumor grafts. Because an injection of CF750-A33scFv-Fc produced high-contrast images in subcutaneous tumor-bearing mice (Figure 3), we further investigated whether the orthotopic tumor grafts could be dissected under the guidance of CF750-A33scFv-Fc-based optical imaging. As shown in Figure 6, after the laparotomy of the mice injected with CF750-A33scFv-Fc, optical imaging demonstrated that a fluorescence signal was predominantly detectable in tumor tissues and the liver. In particular, the excrescent tumor tissue was distinguished from that of the normal colon using the optical imaging system. Finally, the tumor tissue was accurately dissected under the guidance of the optical imaging system. These results suggest that CF750-A33scFv-Fc-based imaging agent might facilitate the surgical removal of colorectal cancer while keeping both patients and surgeons free from radiation.

However, unlike the LS174T tumor grafts, the COLO205 tumor grafts in mice injected with the same amount of CF750-A33scFv-Fc were visualized with low contrast (Figure 5(b)), suggesting a difference between COLO205 and LS174T tumor grafts in uptake of CF750-A33scFv-Fc. To reduce the bias caused by individual differences between mice and batch variations of CF750-A33scFv-Fc, we further used mice bearing both COLO205 and LS174T tumor grafts to evaluate the uptake of CF750-A33scFv-Fc. Scanning the mice and the tissues revealed that the uptake of CF750-A33scFv-Fc by the COLO205 tumor graft was significantly less than that by the LS174T tumor graft (Figure 5(c)). Flow cytometric analysis with FITC-A33scFv-Fc demonstrated that GPA33 was expressed on the surfaces of COLO205 and LS174T cells (Figure 2(a)). Further, there was no obvious difference in either cell line in MFI after incubation with the same amount of FITC-A33scFv-Fc (data not shown). Immunofluorescence histochemistry revealed that even though membrane GPA33 was expressed in both COLO205 and LS174T tumor grafts at a similar level (Figure 2(b)), the LS174T xenografts were hypervascular [32]. The expression of vascular endothelial growth factor (VEGF) in LS174T cells was much higher than that in COLO205, suggesting a difference between the xenografts of LS174T and COLO205 in vascularization [33, 34]. In fact, we found blood vessel-rich areas in LS174T but not in COLO205 tumor xenografts. These different vascularizations may have led to the differences between COLO205 and LS174T tumor xenografts in their delivery of A33scFv-Fc. Consequently, CF750-A33scFv-Fc might be limited in utility for imaging blood vessel-rich colorectal cancer. Still, the injection of a small tumor-homing peptide produced clear images of COLO205 tumor xenografts (data not shown), suggesting that the tissue-penetrating ability of A33scFv-Fc is poor. A33scFv-based diabody or minibody with reduced molecular weight but retained affinity might possess a higher tissue-penetrating ability. Moreover, the tissue penetration of A33scFv-Fc might also be improved by fusion with tissue-penetrating peptides [35].

## Supplementary Material

Brief instruction for Supplementary Figure 1: LS174T cells were incubated with FITC-labelled A33scFv or A33scFv-Fc at indicated concentrations according to the description in materials and methods. Subsequently, the incubated cells were washed with PBS followed by followed by analysis using a flow cytometer. The positive rates and mean fluorescent index (MFI) were calculated and compared. The results demonstrated that the binding rate and mean fluorescence intensity (MFI) of A33scFv-Fc antibody-stained cells were higher than that of cells stained with the A33scFv antibody at the same molar concentration.
Legend for Supplementary Figure 1: Comparison of the binding ability of the A33scFv and A33scFv-Fc for LS174T cells. (a) Flow cytometer analysis of LS174T cells after incubation with A33scFv or A33scFv-Fc at different concentrations (0~20 nM). (b) Comparison of A33scFv and A33scFv-Fc on the positive rates and MFI of LS174T cells incubated with these antibodies.
Brief instruction for Supplementary Figure 2: Mice bearing subcutaneous LS174T xenografts were injected with CF750-A33scFv (equal molar concentration of 100 μg CF750-A33scFv-Fc) and scanned using optical system at different times. The results demonstrated that the tumor uptake of CF750-A33scFv was detected but only low contrast images were documented in 7h post-injection (Supplementary Figure 2a). After the last scanning, mice were sacrificed and the removed organs/tissues were scanned. The uptake rate of CF750-A33scFv was as follows (from high to low): kidney > liver > tumor > stomach. The ratio of tumor to lung, spleen, stomach, colon, small intestine and muscle kidney were 3.56±0.62, 3.391±0.31, 1.63±0.45, 3.23±1.47, 5.37±2.07 and 7.54±2.28, respectively (Supplementary Figure 2b).
Legend for Supplementary Figure 2: CF750-A33scFv-mediated optical imaging of mice bearing subcutaneous LS174T xenografts. (a) Optical images of the mice (n=3) at the indicated time points after intravenously injections with CF750-A33scFv (equal molar concentration of 100 μg CF750-A33scFv-Fc). (b) Tissue distributions of CF750-A33scFv at 7 h post-injection. (c) The fluorescence intensities of CF750-A33scFv in tumor grafts and organs/tissues. LS174T xenografts are indicated with red circle.

## Figures and Tables

**Table 1:** Biodistribution of ^131^I-A33scFv-Fc in nude mice bearing LS174T xenografts (*n* = 3 at each time point).

Tissue	0.08 h	0.5 h	2 h	5 h	8 h	24 h
Blood	20.98 ± 1.87	10.92 ± 2.43	6.36 ± 1.91	2.86 ± 0.14	1.61 ± 0.28	0.68 ± 0.19
Heart	10.36 ± 2.74	5.47 ± 1.17	3.16 ± 0.90	1.41 ± 0.10	0.81 ± 0.14	0.39 ± 0.15
Liver	10.91 ± 1.80	9.52 ± 1.10	6.53 ± 0.78	3.28 ± 0.21	2.68 ± 0.70	2.12 ± 0.88
Spleen	21.62 ± 3.33	16.63 ± 2.96	9.90 ± 1.31	5.03 ± 0.48	3.23 ± 0.22	2.72 ± 1.28
Lung	35.16 ± 10.71	17.48 ± 2.57	8.56 ± 2.49	3.39 ± 0.34	1.66 ± 0.18	0.73 ± 0.23
Kidney	30.35 ± 2.40	27.14 ± 1.54	20.51 ± 1.46	13.50 ± 0.74	11.48 ± 0.96	7.61 ± 2.19
Stomach	11.59 ± 3.28	16.07 ± 2.32	29.63 ± 9.02	11.93 ± 0.64	4.61 ± 1.18	1.28 ± 0.43
Small intestine	8.82 ± 1.72	6.71 ± 1.03	4.36 ± 2.52	2.86 ± 0.96	2.00 ± 1.44	0.47 ± 0.16
Pancreas	9.96 ± 1.17	7.25 ± 0.60	9.37 ± 3.18	4.38 ± 0.44	1.50 ± 0.32	0.29 ± 0.05
Colon	6.39 ± 1.67	3.79 ± 0.87	5.47 ± 1.37	2.07 ± 0.28	0.95 ± 0.19	0.38 ± 0.11
Brain	1.45 ± 0.02	0.85 ± 0.09	0.81 ± 0.20	0.27 ± 0.02	0.15 ± 0.01	0.03 ± 0.01
Muscle	4.00 ± 0.26	2.47 ± 0.12	2.57 ± 0.41	0.98 ± 0.08	0.62 ± 0.12	0.16 ± 0.05
Tumor	4.28 ± 0.37	4.99 ± 0.06	5.45 ± 1.25	4.21 ± 0.29	2.90 ± 0.37	2.03 ± 0.51

%ID/g: the percent of injected dose per gram tissue.
