# Supplementary figures and images for: CF750-A33scFv-Fc-Based Optical Imaging of Subcutaneous and Orthotopic Xenografts of GPA33-Positive Colorectal Cancer in Mice
Source: Biomed Res Int. 2015 May 21;2015:505183. doi: 10.1155/2015/505183 (PMC4454727; doi:10.1155/2015/505183)

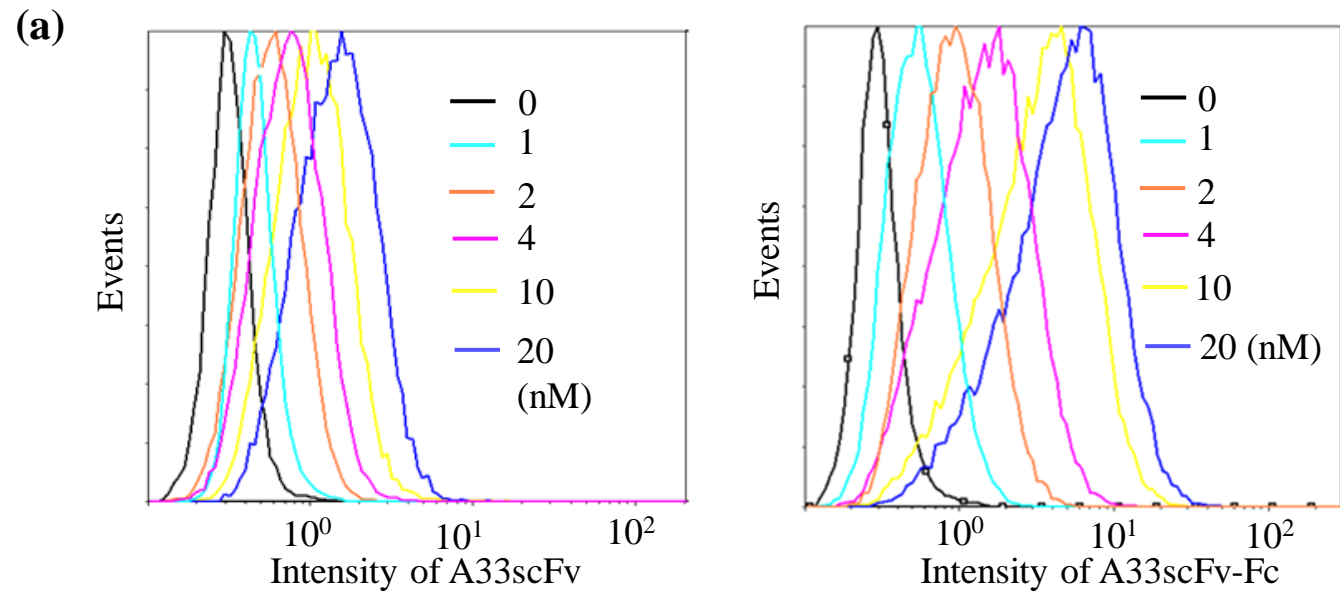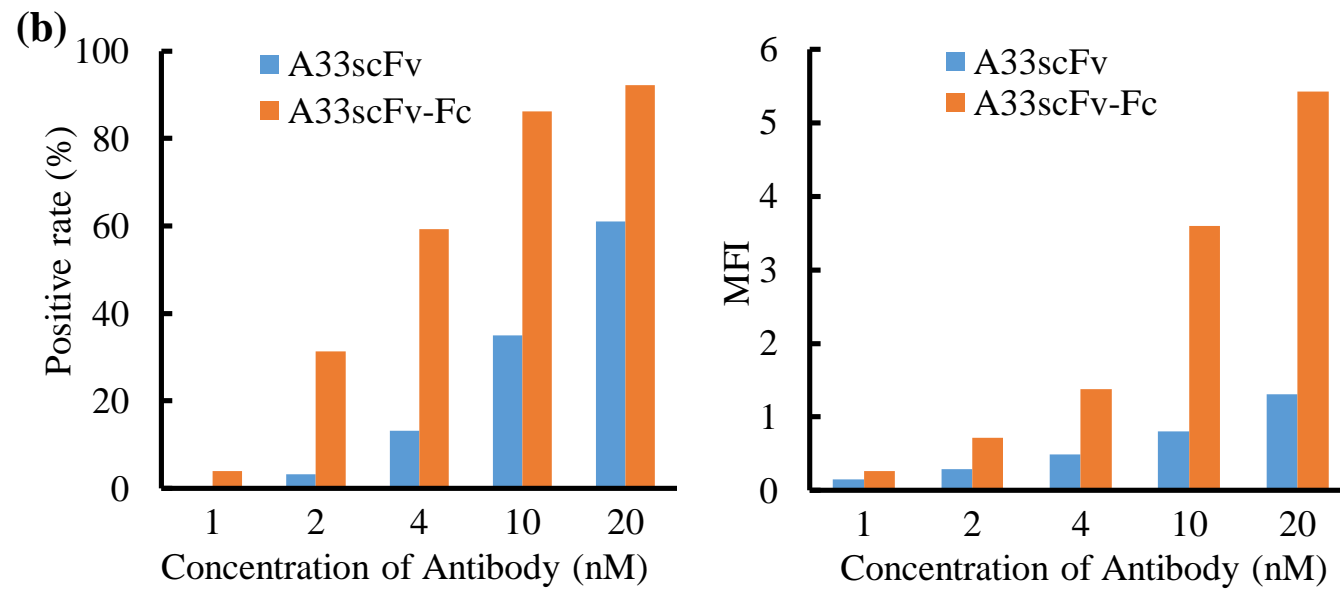

Supplement: Supplementary file 1 — Brief instruction for Supplementary Figure 1: LS174T cells were incubated with FITC-labelled A33scFv or A33scFv-Fc at indicated concentrations according to the description in materials and methods. Subsequently, the incubated cells were washed with PBS followed by followed by analysis using a flow cytometer. The positive rates and mean fluorescent index (MFI) were calculated and compared. The results demonstrated that the binding rate and mean fluorescence intensity (MFI) of A33scFv-Fc antibody-stained cells were higher than that of cells stained with the A33scFv antibody at the same molar concentration. Legend for Supplementary Figure 1: Comparison of the binding ability of the A33scFv and A33scFv-Fc for LS174T cells. (a) Flow cytometer analysis of LS174T cells after incubation with A33scFv or A33scFv-Fc at different concentrations (0~20 nM). (b) Comparison of A33scFv and A33scFv-Fc on the positive rates and MFI of LS174T cells incubated with these antibodies. Brief instruction for Supplementary Figure 2: Mice bearing subcutaneous LS174T xenografts were injected with CF750-A33scFv (equal molar concentration of 100 μg CF750-A33scFv-Fc) and scanned using optical system at different times. The results demonstrated that the tumor uptake of CF750-A33scFv was detected but only low contrast images were documented in 7h post-injection (Supplementary Figure 2a). After the last scanning, mice were sacrificed and the removed organs/tissues were scanned. The uptake rate of CF750-A33scFv was as follows (from high to low): kidney > liver > tumor > stomach. The ratio of tumor to lung, spleen, stomach, colon, small intestine and muscle kidney were 3.56±0.62, 3.391±0.31, 1.63±0.45, 3.23±1.47, 5.37±2.07 and 7.54±2.28, respectively (Supplementary Figure 2b). Legend for Supplementary Figure 2: CF750-A33scFv-mediated optical imaging of mice bearing subcutaneous LS174T xenografts. (a) Optical images of the mice (n=3) at the indicated time points after intravenously injections with CF750 [file 505183.f1.pdf]

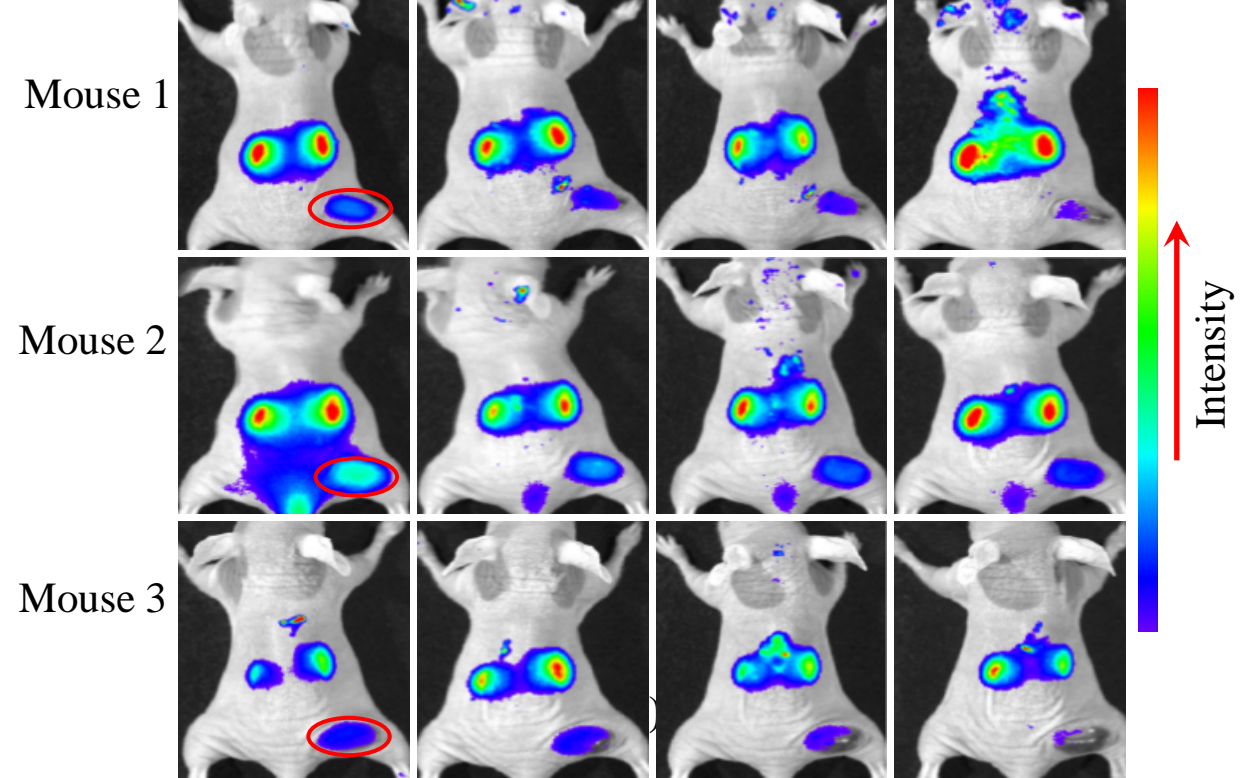

(b)

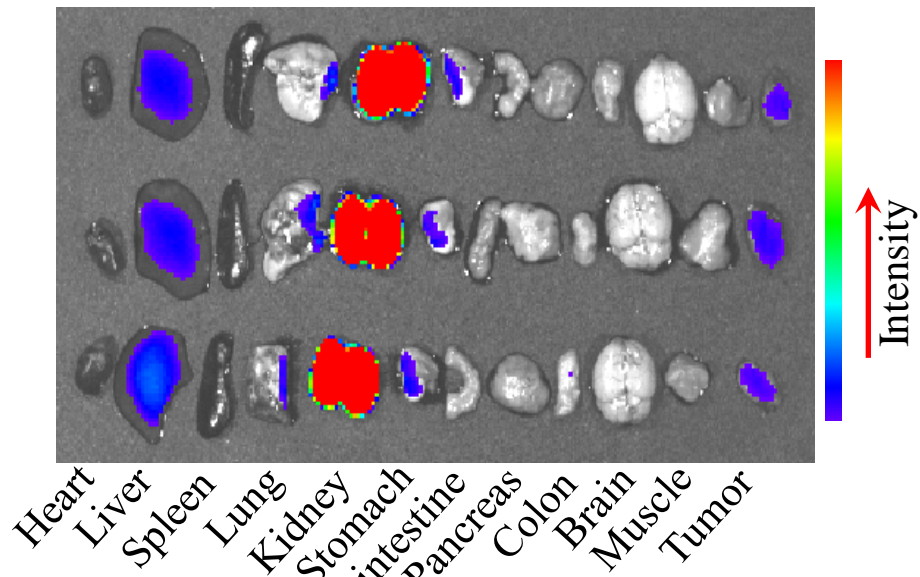

(c)

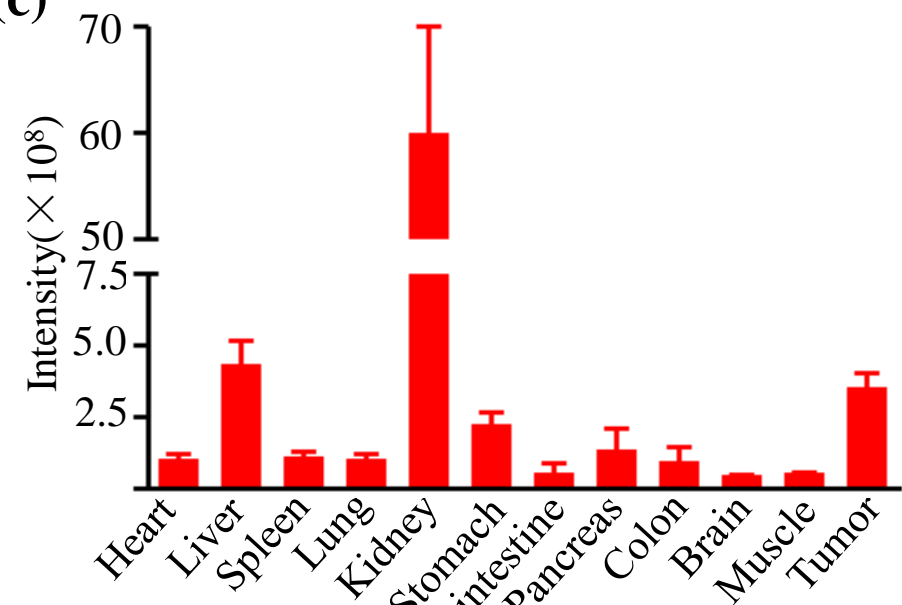

Supplement: Supplementary file 2 [file 505183.f2.pdf]
